# Hea Lthtract, No. 258

**Published:** 1866-09

**Authors:** 


### PHYSIOLOGY OF REPENTANCE.

The religious sentiment of the whole country has experienced a revulsion and a shock recently which, it is to be hoped, will not be repeated while time endures. The model monster, who was recently executed at Philadelphia, for the murder of a confiding family of eight persons within an hour, in the expectation of getting a little money, professed repentance, and a confidence of forgiveness, just as he was swung off by the neck like a dog, and that with a known lie in his mouth: and what makes the matter worse, educated religious advisers did not hesitate to give countenance to the horrible profanation of professing their belief in his sincerity.

Akin to such an absurdity is the workings of the mind when a man wishes to commit a crime; he first persuades himself that the act contemplated is not a crime in this particular case, although as a general rule it is unquestionably so. Men have committed adultery, and then hushed their own consciences by pleading the examples of Abraham and David. Passion, Appetite, Fear—these, when they reign supreme, seem to cloud the intellect, or in some way derange the mental machinery, and, for the time being, prevent its healthful working. It is known by those who have been reared among negro slaves, that they do not believe it wrong to steal from their masters. A lady who had inherited a faithful domestic, to whom was entrusted everything, was so shocked one day in finding her pilfering, that she burst into tears. "La, Missus!" exclaimed the surprised darkey, "you needn't take on so, I'se been doin' sich things all my life." In a professional experience of thirty years at the bedside of the sick and dying, the writer has never known a single case when, in the immediate prospect of death, professions of religious sentiment were for the first time made, that were not repudiated on an unexpected recovery. The truth is a true religious sentiment is the offspring of love, and affection and gratitude to Him whose offspring we are; the semblance a sham piety, arises from threats, fear, compulsion, as was ludicrously exhibited in our little Robbie, when one day, in his seventh year, we being down town, the tall chimney of our dwelling was induced, by a tornado, to make a voyage of discovery through the roof, with a young ocean of water. "What shall we do, mother?" cried the boy, in great terror. "Pray to the Lord, my child.," But Robbie being a minute man, and seeing no signs of the remedy being put in operation by his respected maternal progenitor, exclaimed, with the utmost impatience, the bricks still tumbling in, and the cataract of waters givin no indication of a surcease, "Why don't you do it, then?" and feeling thrown on his own resources, down on his marrow bones he went—"Now I lay me down to sleep." Just as he arose from his spontaneous devotions he observed that his younger sister was following "in the same line;" but looking up through the roof, and seeing the clouds all gone, his whole countenance overspread with joy, exclaimed, "Needn't pray now. Alice, the sun's shining."

Reader, let your piety be prompted by the habitual contemplation of the goodness of God in the sunshine of health and prosperity and a calm life; then, should storms threaten, and adversity come, and sickness waste the health away, you can look it all in the face fearlessly, and feel, as the last life strings are breaking, "I KNOW that my Redeemer liveth;' and at the first blast of the trumpet, which wakes the world to judgment, you will find yourself robed in spotless purity, among the shining ones.

